# Persistence of Zika virus in conjunctival fluid of convalescence patients

**DOI:** 10.1038/s41598-017-09479-5

**Published:** 2017-09-11

**Authors:** Jeslin J. L. Tan, Praveen K. Balne, Yee-Sin Leo, Louis Tong, Lisa F. P. Ng, Rupesh Agrawal

**Affiliations:** 1 Singapore Immunology Network, Agency for Science, Technology and Research (A*STAR), Singapore, Singapore; 2 National Healthcare Group Eye Institute, Tan Tock Seng Hospital, Singapore, Singapore; 3 Singapore Eye Research Institute, Singapore, Singapore; 4 Yong Loo Lin School of Medicine, National University of Singapore, Singapore, Singapore; 5 Institute of Infectious Disease and Epidemiology, Tan Tock Seng Hospital, Singapore, Singapore; 6 Singapore National Eye Center, Singapore, Singapore; 7 Duke-NUS Medical School, Singapore, Singapore; 8 Institute of Infection and Global Health, University of Liverpool, Liverpool, United Kingdom

## Abstract

A widespread epidemic of Zika fever, caused by Zika virus (ZIKAV) has spread throughout the Pacific islands, the Americas and Southeast Asia. The increased incidences of ocular anomalies observed in ZIKAV-infected infants and adults may be associated with the rapid spread of ZIKAV. The objective of this study was to check if ZIKAV could be detected in human tears after the first week of infection. Twenty-nine patients with PCR confirmed ZIKAV infection during the Singapore August 2016 ZIKAV outbreak were enrolled for the study. Detection and quantification of ZIKAV RNA was performed on conjunctival swabs collected from both eyes of these patients at the late convalescent phase (30 days post-illness). Efficiency of viral isolation from swab samples was confirmed by the limit of detection (as low as 0.1 PFU/µL, equivalent to copy number of 4.9) in spiked swabs with different concentrations of ZIKAV (PFU/µL). Samples from three patients were found positive by qRT-PCR for ZIKAV and the viral RNA copy numbers detected in conjunctival swabs ranged from 5.2 to 9.3 copies respectively. ZIKAV could persist in the tears of infected patients for up to 30 days post-illness, and may therefore possess a potential public health risk of transmission.

## Introduction

Zika virus (ZIKAV) is a mosquito-borne *flavivirus* which was first isolated in 1947 from a sentinel rhesus macaque in Uganda^1^. ZIKAV has remained in relative obscurity for nearly 60 years until the unprecedented 2007 outbreak in the Western Pacific island of Yap Federated States of Micronesia^2^. This was then followed by a larger epidemic in French Polynesia in 2013 and 2014^3^. Subsequent smaller outbreaks occurred in the Pacific islands from 2014 to 2016^4^. ZIKAV was introduced into Brazil in 2015, and within that year, it spread rapidly throughout the Americas^5, 6^. Reports of imported and autochthonous ZIKAV infections have also been observed in South East Asia^7^ where arboviral diseases are very common^8, 9^. Singapore reported its first case of local ZIKAV transmission on 27 August 2016^10^.

ZIKAV infection is rarely life threatening, manifesting typically as short-lived fever, nonspecific rash, and joint pain, with many patients being completely asymptomatic^2, 11^. However the re-emergence of ZIKAV has been associated with severe neurological complications: Guillain-Barré syndrome (GBS) in French Polynesia^12, 13^ and microcephaly in Brazil^14–16^. Although the full extent of ZIKAV ophthalmologic manifestations is unclear, ocular developmental anomalies have been reported in infants with microcephaly in Brazil^11, 17–19^. To date, two cases of ZIKAV-associated uveitis in adult patients have also been reported^20, 21^. The occurrence of uveitis suggests a non-infective inflammatory response to systemic or ocular infection with ZIKAV, or it could actually represent an actual viral infection such as choroiditis or vitritis. In an earlier report from China, ZIKAV was found on swabs from the conjunctiva (superficial mucosa covering the globe)^22^. However, it was unclear if ZIKAV could be localized after the first week of infection.

In this report, we describe the detection of ZIKAV RNA from conjunctival swab samples of laboratory-confirmed ZIKAV cases collated during the 2016 ZIKAV outbreak in Singapore.

## Methods

### Participants

This study included conjunctival swab samples obtained from patients admitted to the Communicable Disease Centre at Tan Tock Seng Hospital from 29^th^ August to 15^th^ November 2016. Conjunctival samples were collected from both eyes of study subjects by pulling the lower eyelid down and drawing the tip of the sterile swab (FLOQSwabs®, Copan Flock Technologies, Brescia, Italy) gently across the inside of the lower eyelid, at visit 3 which was at day 30 post-illness. Samples were processed on the same day of collection and stored at −80 °C until use. These patients were previously confirmed to be ZIKAV-infected by quantitative reverse-transcription polymerase chain reaction (qRT-PCR) performed on serum and/or urine samples obtained during their first visit to the clinic^23, 24^.

### Standard Protocol Approvals, Registrations and Patient Consents

Written informed consent was obtained from all participants in accordance with the tenets of the declaration of Helsinki for human research. The study protocol was approved by the SingHealth Centralized Institutional Review Board (CIRB Ref: 2016/2219) and National Healthcare Group Institutional Review Board.

### Virus Isolate

The ZIKAV strain H/PF/2013 used in this study was originally isolated from a French patient returning from French Polynesia during the 2013 Zika outbreak^25^. After passages in Vero-E6 cultures, virus stocks were washed, and precleared by centrifugation before storing at −80 °C. All virus stocks were titered by plaque assay and quantified by qRT-PCR as previously described^26^.

### Spiked Samples for Extraction Performance

ZIKAV was first serial diluted before being added into aliquots of Minimum Essential Medium (MEM) (Sigma-Aldrich, Munich, Germany) to cover 10^−4^ to 10^3^ PFU/µL. Clean swabs (FLOQSwabs®) were immersed into respective ZIKAV dilutions. Each spiked swab was then immersed in 400 µL MEM respectively and pulse-vortexed for 1 minute. Viral RNA was subsequently isolated from 140 µL viral-MEM mixture using QIAamp Viral RNA Mini Kit (Qiagen, Hilden, Germany). Spiked experiments were repeated twice for inter-experimental reproducibility.

### ZIKAV Real-Time RT-PCR

Both right and left conjunctival swabs from each patient were immersed in 400 µL of MEM. Re-suspension of patient swabs (in MEM) and subsequent viral RNA extraction were carried out as described earlier. Quantification by ZIKAV real-time reverse-transcription polymerase chain reaction (RT-PCR) of ZIKAV RNA for all samples was performed by one-step TaqMan real-time RT-PCR (QuantiTect Probe RT-PCR kit, Qiagen) using primers and probes described previously^24^. Briefly, all reactions were performed in a final volume of 25 μL with 5 μL of RNA, 400 nM and 200 nM of primers and probe respectively, 0.25 μL of RT enzyme and 12.5 μL of QuantiTect Probe RT-PCR master mix in 7500 Real Time PCR System (Applied Biosystems, Singapore). The following cycling conditions were used: 50 °C for 30 minutes, 95 °C for 10 minutes, followed by 45 cycles of 94 °C for 15 seconds and 60 °C for 1 minute. Validation, sensitivity and specificity of the qRT-PCR assay have been previously reported and demonstrated^24^. Specificity of the assay was evaluated by testing the following viral RNAs, all of which yielded negative results: Dengue serotype 1–4 viruses, West Nile virus, St. Louis encephalitis virus, yellow fever virus, Powassan virus, Semliki Forest virus, o’nyong-nyong virus, chikungunya virus, and Spondweni virus^24^. The qRT-PCR assay has also been used in numeral studies for ZIKAV RNA quantification in patient samples^27–29^, demonstrating its specificity, feasibility and reproducibility. RNA transcripts of ZIKAV strain H/PF/2013 ranging from 10^9^ to 1.0 copies were tested in quadruplicates to construct the standard curve for estimating the copy number of ZIKAV RNA in swab samples.

## Results

Of the 29 ZIKAV-infected patients in the study, majority were Chinese (n = 22, 75.9%), followed by Malay (n = 3, 10.3%), Indian (n = 3, 10.3%) and one from Bangladesh (Table 1). Male to female ratio was 16:13. Patient age ranged from 21 to 61 years (mean, 40). Rash (n = 28, 96.6%) and fever (n = 20, 69.0%) were the most common presenting symptoms at hospital admission. Conjunctivitis (red eye) was the ocular presentation observed in eight patients (27.5%) (Table 1). All patients with conjunctivitis experienced both watering and non-purulent discharge with discomfort. In addition, three (10.3%) patients suffered from pain in the eyes (Table 1). There was no complaint of decrease in vision in any of the patients. However, a complete ocular examination including visual acuity, fundus evaluation and conjunctival swabs could not be performed on the patients at time of first disease presentation.

**Table 1:** Quantitative real-time RT-PCR of conjunctival samples from ZIKAV positive patients.

Patients		Demographics			Symptoms^a^				ZIKAV VL		Left Eye^b^			Right Eye^b^		
No	ID	Age	Gender	Ethnic	Rash	Fever	Conjunctivitis	Retro-Orbital pain	Blood VL^b^	Urine VL^b^	LE Ct value^c^	LE Copies^e^	LE Detection Result^d^	RE Ct value^c^	RE Copies^e^	RE Detection Result^d^
**1**	**36**	**47**	**Female**	**Chinese**	**+**	**+**	**+**	**+**	**11.6**	**0.0**	**ND**	**NIL**	**−**	**37.7**	**9.3**	**+**
**2**	**74**	**22**	**Female**	**Malay**	**+**	**−**	**−**	**−**	**4.2**	**0.0**	**ND**	**NIL**	**−**	**38.6**	**5.2**	**+**
**3**	**84**	**52**	**Female**	**Indian**	**+**	**−**	**+**	**−**	**0.0**	**0.0**	**ND**	**NIL**	**−**	**38.5**	**5.3**	**+**
4	14	28	Female	Chinese	+	+	**−**	**−**	0.0	0.0	ND	NIL	**−**	ND	NIL	**−**
5	15	33	Female	Chinese	+	+	**−**	+	3.5	0.0	ND	NIL	**−**	ND	NIL	**−**
6	27	21	Female	Chinese	+	+	**−**	+	0.0	0.0	ND	NIL	**−**	ND	NIL	**−**
7	30	56	Male	Chinese	+	+	+	**−**	0.0	0.0	ND	NIL	**−**	ND	NIL	**−**
8	33	26	Male	Chinese	+	+	**−**	**−**	42.4	0.0	ND	NIL	**−**	ND	NIL	**−**
9	34	42	Male	Chinese	+	+	**−**	**−**	0.0	0.0	ND	NIL	**−**	ND	NIL	**−**
10	35	28	Male	Bangladesh	+	+	**−**	**−**	0.0	0.0	ND	NIL	**−**	ND	NIL	**−**
11	43	43	Female	Chinese	+	**−**	**−**	**−**	3.5	0.0	ND	NIL	**−**	ND	NIL	**−**
12	50	39	Female	Malay	+	+	+	**−**	3.4	0.0	ND	NIL	**−**	ND	NIL	**−**
13	53	30	Male	Chinese	+	+	**−**	**−**	23.2	0.0	ND	NIL	**−**	ND	NIL	**−**
14	54	37	Female	Chinese	+	+	**−**	**−**	10.2	0.0	39.0	NIL	**−**	ND	NIL	**−**
15	55	29	Male	Chinese	+	+	**−**	**−**	17.3	0.0	ND	NIL	**−**	ND	NIL	**−**
16	56	37	Male	Chinese	+	+	**−**	**−**	0.0	49.0	ND	NIL	**−**	ND	NIL	**−**
17	61	36	Female	Chinese	+	+	**−**	**−**	4.1	0.0	ND	NIL	**−**	ND	NIL	**−**
18	68	35	Male	Chinese	**−**	**−**	**−**	**−**	0.0	0.0	ND	NIL	**−**	ND	NIL	**−**
19	69	53	Female	Chinese	+	**−**	**−**	**−**	3.5	0.0	ND	NIL	**−**	ND	NIL	**−**
20	70	58	Female	Indian	+	**−**	**−**	**−**	0.0	0.0	ND	NIL	**−**	ND	NIL	**−**
21	72	60	Male	Chinese	+	+	+	**−**	0.0	86.9	ND	NIL	**−**	ND	NIL	**−**
22	73	21	Female	Malay	+	+	**−**	**−**	0.0	0.0	ND	NIL	**−**	ND	NIL	**−**
23	75	54	Male	Indian	+	**−**	**−**	**−**	30.7	0.0	ND	NIL	**−**	ND	NIL	**−**
24	76	26	Female	Chinese	+	**−**	**−**	**−**	0.0	0.0	ND	NIL	**−**	ND	NIL	**−**
25	80	52	Male	Chinese	+	+	+	**−**	0.0	0.0	ND	NIL	**−**	ND	NIL	**−**
26	83	33	Male	Chinese	+	+	+	**−**	0.0	0.0	ND	NIL	**−**	ND	NIL	**−**
27	86	41	Female	Chinese	+	+	**−**	**−**	0.0	0.0	ND	NIL	**−**	ND	NIL	**−**
28	87	50	Male	Chinese	+	**−**	+	**−**	0.0	0.0	ND	NIL	**−**	ND	NIL	**−**
29	88	61	Female	Chinese	+	+	**−**	**−**	37.8	0.0	ND	NIL	**−**	ND	NIL	**−**

ND, Not detectable; Ct value, crossing threshold; dpi, Days Post Illness onset; VL, viral load; LE, left eye; RE, right eye.

Patients with positive ZIKAV detection in one or both eyes are in bold.

^a^+, Yes −, No.

^b^Sample tested at convalescence (30 dpi).

^c^Ct values < 39.0 are positive.

^d^+, positive; −, negative.

^e^Estimated by testing quantitated dilutions of ZIKAV RNA transcripts.

Sample isolation efficiency was first investigated to assess the resultant efficiency of the extraction method using spiked swabs. The estimated PFU on the ZIKAV-spiked swab compared to the viral load derived from qRT-PCR was shown in (Table 2). The estimated detection threshold was 0.1 PFU, detected at approximately 4.9 copies after isolation.

**Table 2:** Viral RNA Isolation efficiency and qRT-PCR sensitivity using ZIKAV spiked swabs.

Spiked concentrations, PFU/µL	ZIKAV Real Time qRT-PCR	
	Ct value	Viral load, copies^a^
1,000	25.7	2.8E + 04
100	29.9	1.6E + 03
10	33.5	1.5E + 02
1.0	37.3	1.2E + 01
0.1	38.7	4.9E + 00
0.01	ND	0
Clean swab only	ND	0

ND, Not detectable; Ct value, crossing threshold; ^a^Estimated by testing quantitated dilutions of ZIKAV RNA transcripts.

After evaluating the efficiency of the isolation method, patient samples were processed following the established workflow. ZIKAV RNA was detected in conjunctival swabs of three infected patients at the late convalescent phase of the disease (30 days post-illness). The estimated ZIKAV RNA loads in the conjunctiva of these patients ranged from 5.2 to 9.3 copies (Table 1). Two of the three patients suffered from conjunctivitis, with one experiencing retro-ocular pain.

## Discussion

In this study, we described the direct isolation and detection of ZIKAV RNA from conjunctival swab samples. Although detection of ZIKAV from conjunctiva has been previously reported^22^, presence of ZIKAV RNA in conjunctiva at the late convalescent phase of the disease was not known. ZIKAV RNA, however, was detected in one of the two eyes of virus-infected patients at 30 days post-illness. Due to the non-invasiveness of conjunctival swabs, testing of tear samples could be useful for diagnosis and evaluation of disease status.

Importantly, we also investigated the minimum amount of ZIKAV RNA (after swab sample isolation) required for amplification. The efficiency of such a detection assay is often dependent on the efficiency of the nucleic acid extraction from clinical specimens^30, 31^. Some extraction methods may interfere with the PCR reaction^32^. Efficiency of the isolation method was demonstrated by amplicon yield in the subsequent qRT-PCR. The ability of the isolation to yield ZIKAV RNA loads within the detection window of the qRT-PCR from a low-concentrated spiked swab substantiates the validity of the workflow. Moreover, our detection range was broad as shown by the spiked swabs. This is clinically important because ZIKAV viral loads observed in swab samples of ZIKAV-infected patients ranged from approximately 2.0 to 1788 copies/µL^22^.

It has been reported that ZIKAV RNA was still detected in virus-infected patients’ blood up to 11 days post-illness^24^, in urine and semen at 80 days^33^, and in tears at 7 days post-illness^22^. Our study demonstrated the longest persistence of ZIKAV RNA in tears at 30 days post-illness. Collection of swab samples at the late convalescent phase of disease could be a possible reason for the low viral RNA copy numbers observed. Despite the late collection, sensitivity was not compromised while the other report^22^ indicated only up to 7 days post-fever onset. To note, two of the patients still had detectable viral RNA copies in blood (patient 036 = 11.6 copies, patient 074 = 4.2 copies) during the time of tears collection at 30 days post-illness. Interestingly, viral load in blood for patient 036 was moderately high at 19.8 and 272.7 copies during the acute (5 days post-illness) and early convalescent (13 days post-illness) phases of the infection respectively, that persisted till late convalescence. Presence of ZIKAV RNA (81.6 copies) was also detected in the urine sample of patient 036 at early convalescence. The persistence of ZIKAV in both blood and urine could possibly explain for the presence of ZIKAV RNA in the conjunctival fluid. The qRT-PCR cycle threshold below the cut off showed that ZIKAV was present at the time (30 days post-illness) after patient 036 had recovered. Future studies with larger number of positive specimens are warranted to further establish the mechanisms of ZIKAV-induced ocular disease and viral persistence in the eye.

Nevertheless, the important clinical significance of this report is that ocular transmission of the ZIKAV through contact with ocular discharge is potentially possible even up to the late convalescent phase of ZIKAV infection. Transmission of ZIKAV through tears, although uncommon, was highlighted as a possible mode of transmission in an earlier case study^34^. ZIKAV infection in another individual who had close contact with a ZIKAV-infected patient with high viremia was also reported^35^. Similar findings were reported in a dengue patient^36^ and also in *in vivo* experiments^37^. These findings suggest that the spectrum of those at risk of ZIKAV infection may be broader than previously recognized. It is essential that health care personnel and community contacts are aware of the risks of virus infection and apply standard precautions while caring for ZIKAV-infected patients. Few systematic studies have examined the presence of ZIKAV in aqueous humor obtained from one and/or both eyes of infected patients after the onset of illness, so the incidence and clinical manifestations of ZIKAV ocular complications are uncertain. Interestingly, one of the patients with positive ZIKAV detection in the conjunctiva did not have any evidence of conjunctival inflammation or ocular pain at time of tears collection. This is worrying as this phenomenon suggests that a lack of clinical signs in the eye may not preclude infectious potential from the eye. Eventual investigations should determine if ZIKAV affects uveitis and other structures of the eye, such as the retinal vasculature, and determine the immunological responses that produce these clinical manifestations.

This case highlights a complication of ZIKAV with important implications for public health. It is important to assure that samples of conjunctivae and tears are tested negative for ZIKAV in patients to ensure no risk of virus spread through casual contact.
